# The Dynamic Associations of Social and Intellectual Activity With Frailty Trajectory in Middle-Aged and Older Adults in China: Nationwide Longitudinal Study

**DOI:** 10.2196/80152

**Published:** 2025-12-15

**Authors:** Fei Xu, Xinlei Miao, Shuang Liu, Yangxuan He, Meng Li, Jiayi Deng, Yilin Zhu, Jingshan Jiang, Song Leng

**Affiliations:** 1 Department of Health Management Center, The Second Affiliated Hospital of Dalian Medical University, No.467, Zhongshan Road, Shahekou District, Dalian, Liaoning, 116023, China, 86 17709875677, 86 0411-84686593; 2 School of Public Health, Dalian Medical University, Dalian, Liaoning, China

**Keywords:** frailty, social activity, intellectual activity, trajectory modelling, CHARLS, China Health and Retirement Longitudinal Study

## Abstract

**Background:**

Research on the effects of social and intellectual activities on frailty remains limited, particularly longitudinal studies examining how these activities influence the dynamic progression of frailty over time. We aimed to examine this association in a nationally longitudinal study.

**Objective:**

This study aimed to investigate the relationship between social and intellectual and frailty trajectories in the middle-aged and older Chinese population. Specifically, it sought to identify distinct frailty trajectories and quantify the longitudinal protective effects of social and intellectual activities against adverse frailty progression, while exploring differential impacts across age, sex, and residence subgroups.

**Methods:**

This study used data from the China Health and Retirement Longitudinal Study and group-based trajectory modeling identified frailty trajectories over seven years. Frailty was assessed using a 38-item frailty index. The frequency of social activities and intellectual activity were quantified separately. Multivariate ordinal logistic regression analyzed associations between activity frequencies and trajectory membership.

**Results:**

Three different trajectories of frailty were identified in this study: “low progressive trajectories” (n=7208, 65.8%), “moderate progressive trajectories” (n=3061, 28.5%), and “high progressive trajectories” (n=609, 5.7%). Compared to nonparticipants, frequent social activity participation reduced the likelihood of transitioning to the “moderate progressive” trajectory (odds ratio [OR] 0.84, 95% CI 0.75‐0.94; *P*=.004). Intellectual activity engagement lowered risks of both “moderate” (OR 0.77, 95% CI 0.63‐0.94; *P*=.01) and “high progressive” trajectories (OR 0.63, 95% CI 0.40‐0.99; *P*=.04). Subgroup analyses revealed differential effects by age, sex, and residence.

**Conclusions:**

This study confirms the existence of heterogeneous long-term frailty trajectories among middle-aged and older Chinese adults, social and intellectual activities significantly mitigate frailty progression in Chinese middle-aged and older adults. Regular participation in structured social and intellectual activities can effectively delay frailty development in this population.

## Introduction

Frailty is a complex age-related clinical condition characterized by a decline in physiological capacity across multiple organ systems, leading to increased susceptibility to stressors [1]. As an emerging global health burden, frailty has significant implications for clinical practice and public health [2]. Globally, systematic assessments indicate that the prevalence of frailty among community-dwelling adults aged 65 years and older varies widely, ranging from 4.0% to 59.1% [3]. In a follow-up cohort study involving frail older adults across 28 countries, the incidence of comorbidities associated with frailty was reported to be 43.4 per 1000 person-years [4]. Frailty imposes a substantial burden on national health care utilization and expenditure costs. Given the increasing prevalence and growing harm caused by frailty, identifying it and implementing interventions to slow its progression is crucial for the healthy [5,6].

As a multifactorial syndrome, frailty is influenced by a range of modifiable risk factors, among which, physical, social, and intellectual activities have garnered significant research interest for their potential protective roles. Numerous studies have confirmed that subjects with low social support or social engagement, measured by social frequency, are more likely to exhibit a rapid increase in frailty trajectory, often validated by professional tools such as the Fried phenotype or frailty indices [7-10]. Similarly, a prospective cohort study suggested that frequent intellectual activities—such as reading, playing games, or other cognitively stimulating pursuits—have been identified as a key modifiable factor for mitigating frailty [11]. While these studies underscore the potential protective role of social and intellectual activities, many focus primarily on frailty status or onset rather than its long-term dynamic progression. Furthermore, the specific correlation between the frequency of these activities and distinct longitudinal frailty trajectories remains underexplored in the context of an aging society, particularly within large national cohorts like CHARLS (China Health and Retirement Longitudinal Study).

In summary, this study aims to evaluate the association between social/intellectual activities and distinct frailty trajectories in middle-aged and older adults. The findings may inform strategies for preventing frailty onset and improving outcomes in individuals with frailty.

## Methods

### Study Population and Design

The analyses were performed based on the CHARLS. CHARLS is a longitudinal survey of inhabitants in mainland China aged 45 and older that provides information such as socioeconomic position and health status. It covers 17,708 participants from 150 counties of 28 provinces in China [12]. The data on socioeconomic status, lifestyles, medications, health status, and functioning assessments were collected. Details on the study design, sampling procedure, and details of the surveys are available in its report [12].

Our study used four waves of data (2011‐2018). Participants were restricted to individuals aged ≥45 years who had attended the “health status and function” module at baseline, yielding an initial sample of 17,596. Exclusions comprised 3048 individuals: 497 were aged <45 years, 2436 lacked frailty composite indicator data, and 115 had missing social or intellectual activity data at baseline. During follow-up between 2011 and 2013, 2557 individuals were excluded due to attrition (including death, withdrawal, or loss of contact), while 878 newly eligible individuals were enrolled in 2013. A further 1991 participants were excluded between 2013 and 2018 due to loss to follow-up. A comparison of baseline characteristics between the final analytical cohort and participants lost to follow-up is provided in Multimedia Appendix 1.

### Ethical Considerations

Ethical approval for the China Health and Retirement Longitudinal Study (CHARLS) was obtained from the Biomedical Ethics Review Committee of Peking University (Approval Number: IRB00001052-11015). Written informed consent was secured from all participants prior to enrollment. No direct compensation was provided to participants for this survey. For this secondary analysis, we used fully anonymized and deidentified data from CHARLS.

### Assessment of Frailty

Frailty was assessed using a frailty index (FI), constructed following a standardized procedure [13]. The detailed method for the calculation of FI was reported in previously published studies [14,15]. Briefly, 38 deficit variables relevant to frailty were selected, including impairments in activities of daily living (ADL) and instrumental activities of daily living (IADL), covering 11 tasks such as personal hygiene, dressing, and financial management. The index also incorporated limitations in physical function (9 items), chronic diseases (9 items), psychological health markers (5 items), and subjective measures (eg, self-rated health).

Each variable was scored dichotomously (0=no deficit, 1=deficit present). The FI score was derived by summing all deficits and dividing by the total number of variables. Frailty was defined as FI ≥ 0.25. Full variable definitions and methodological details are provided in Multimedia Appendix 2.

### Assessment of the Frequencies of Social and Intellectual Activities

In the CHARLS survey, participants reported their social and intellectual activities over the preceding month. Social activities, which included four items, were defined as: interacting with friends; attending sports/social clubs; participating in community organizations; and engaging in voluntary or charity work. Intellectual activities, comprising four items, were defined as: playing Ma-jong, playing chess, playing cards, or going to the community club; attending educational courses; investing in stocks; and browsing the internet. Each activity was assigned scores based on the number of participants (Multimedia Appendix 3). The frequency of participants in social activities and intellectual activities was counted separately, categorized into three groups: ≥3 (frequent participation), 1‐2 (nonregular participation), and 0 (no participation) [11].

### Covariates

Based on previous research, several sociodemographic and health-related covariates were included in the study. Sociodemographic covariates comprised age, sex, marital status, location of residence, educational attainment, and retirement status. Health-related covariates included current drinking, current smoking, physical activity levels, and inpatient care. Current drinking and smoking statuses were assessed via self-report using the following questions: “Do you currently smoke?” and “Do you currently drink alcohol?” Marital status was categorized as “married” or “unmarried.” Location of residence was classified as “rural” or “urban,” and educational attainment was grouped into three categories: “primary school or below,” “middle school,” and “high school or above.” Physical activity levels were categorized based on participation frequency as “inactivity,” “low-intensity activities,” “moderate activities,” or “vigorous activities.” Inpatient care was evaluated by the question: “Have you received inpatient care in the past year?” Retirement status was determined by the question: “Are you currently retired?”

### Statistical Analysis

Continuous variables were assessed for normality and are presented as median (25th–75th percentiles) due to their non-normal distribution. Group comparisons were performed using the Kruskal-Wallis test, Pearson’s *χ*² test, Wilcoxon rank-sum test, and Fisher exact test, with false discovery rate correction for multiple comparisons. Categorical variables are summarized as frequencies and percentages, and group differences were assessed using *χ*² tests.

The Group-Based Trajectory Modeling (GBTM) approach was employed to investigate frailty trajectories in middle-aged and older adults. This method applies finite mixture modeling to identify observations with similar temporal developments and assign them to distinct trajectory groups. The optimal trajectory groups were selected based on the following criteria to the greatest extent possible [16]: (1) smaller absolute Bayesian Information Criterion (BIC) values; (2) larger BIC changes; (3) average posterior probability (APP)>0.7; (4) odds of correct classification (OCC) >5; (5) class proportions ≥5%; (6) relative entropy (Ek) is close to 1; (7) parsimony; and (8) interpretability of group patterns.

Subsequently, a multinomial logistic regression model was utilized to estimate the associations between social and intellectual activities and frailty trajectory patterns. Adjusted odds ratios (OR) and corresponding 95% CI are reported.

The multivariable-adjusted model incorporated the following covariates: age at baseline (continuous), sex (male vs female), education (primary school and below, middle school, high school and above), marital status (married vs others), smoking status (yes vs no), alcohol consumption (yes vs no), location of residence (rural vs. city), physical activity levels (inactivity, low-intensity activities, moderate activities, vigorous activities), inpatient care history (yes vs no), and retirement status (yes vs no). The benefits of social and intellectual activities may vary distinctly by subgroup. Sex-based differences in social roles [17], age-related transitions in health status [18], and China’s pronounced rural-urban disparities in infrastructure are all posited as key modifiers of these associations [19]. Thus, stratified analyses were additionally performed in separate models by age groups (<65 y and ≥65 y), sex (male vs female), and location of residence (rural vs city).

Two sensitivity analyses were conducted to assess the robustness of the associations between social/intellectual activities and frailty. First, the 878 newly enrolled participants in 2013 were excluded; second, frailty was stratified into three categories: nonfrailty, prefrailty, and frailty, to investigate its association with social and intellectual activities.

## Results

### Baseline Characteristics

**Figure 1.**
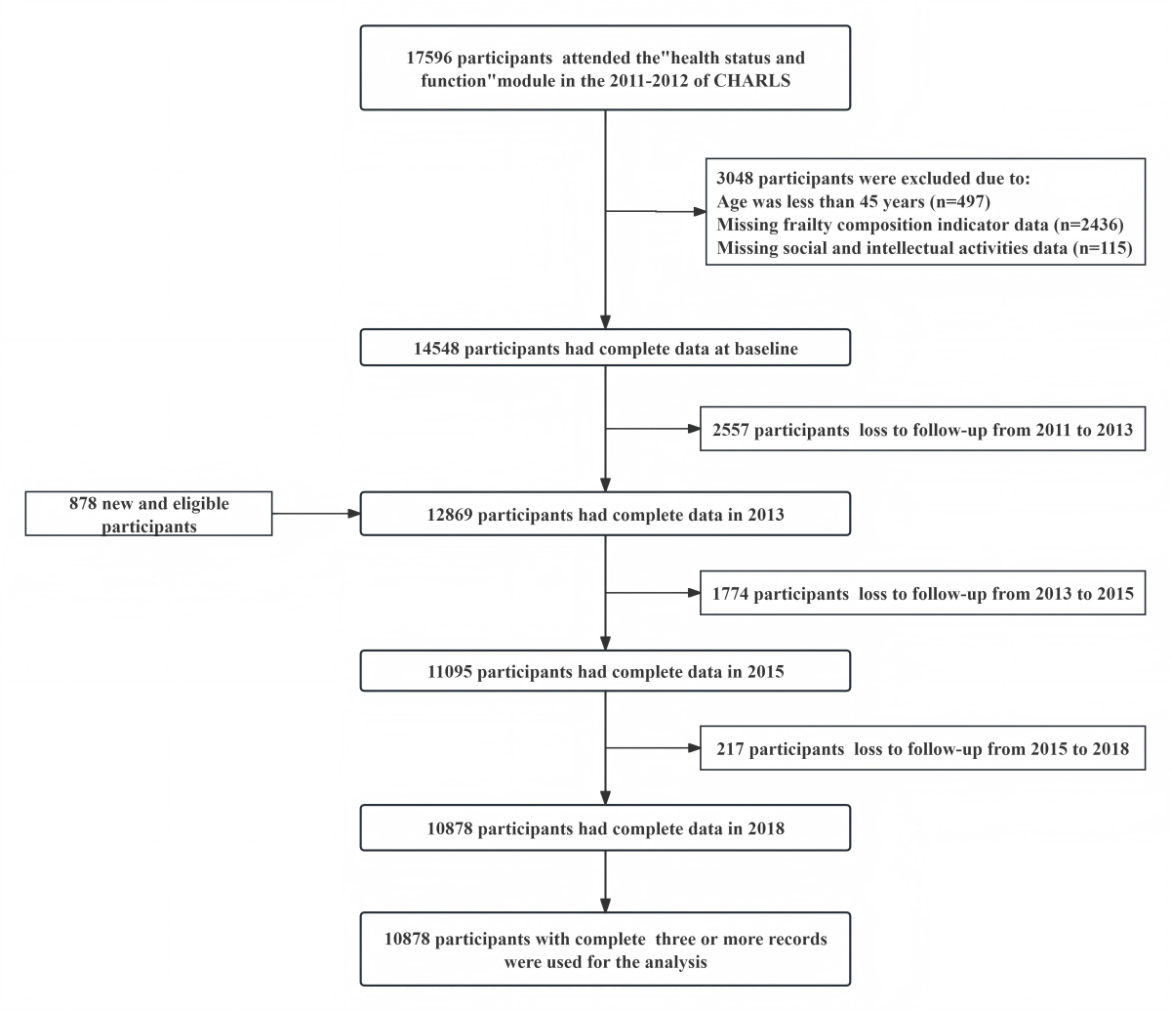
Schematic representation of the inclusion and exclusion process for 10,878 participants.

Of the initial 17,596 participants, a total of 10,878 individuals with three or more complete records comprised the final analytical cohort for trajectory modeling. The detailed flow of participant inclusion and exclusion is presented in Figure 1.

A total of 10,878 participants were included in the study, with 52.0% (n=5655) being female. Among them, 19.7% (n=2143) had a social activities score≥ 3, and 6.1% (n=662) had an intellectual activities score≥ 3. Most participants resided in rural areas, were married, had a primary school education or below, reported physical inactivity, and had retired (*P*<.001).

### Estimated Frailty Trajectories

**Figure 2.**
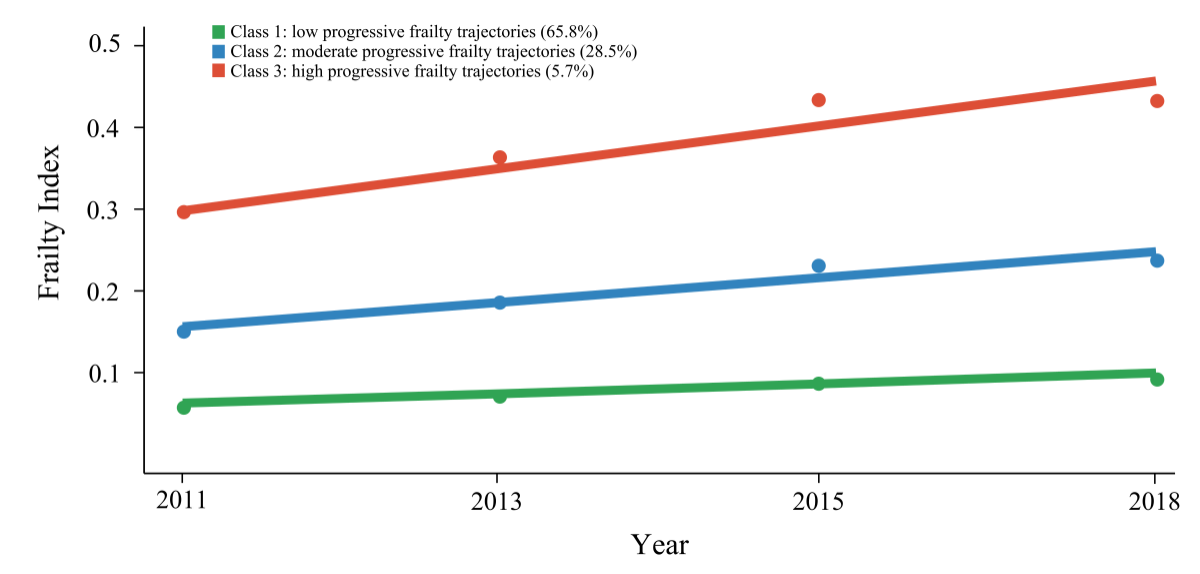
Mean trajectories of frailty scores by increasing age among middle-aged and older adults.

The trajectories model, consisting of three different trajectories, showed the best fit using the BIC, Akaike Information Criterion, APP, and interpretability in the trajectories analysis (Multimedia Appendix 4). Consequently, the GBTM with three trajectories was selected as the optimal model. Figure 2 illustrates the trajectories labeled as: (1) “low progressive” (n=7208, 65.8%), (2) “moderate progressive” (n=3,061, 28.5%), and (3) “high progressive” (n=609, 5.7%). The frailty function trajectories across the three groups are depicted in Figure 1. The “low progressive” group, comprising the majority of participants (65.8%), exhibited minimal FI values that remained consistently low throughout the observation period. The “moderate progressive” group (28.5%) showed gradual progression toward mild frailty over seven years, while the “high progressive” group (5.7%) displayed rapid FI escalation within approximately four years, followed by a plateau.

Comparative analysis of baseline characteristics across trajectory groups revealed that participants in the “high progressive” group were older, more likely to be female, married, rural residents, former smokers or alcohol consumers, and had higher educational attainment (high school or above). Additionally, this group exhibited higher rates of physical inactivity, hospitalization history, and reduced participation in social and intellectual activities compared to the “low progressive” group. Detailed distribution of baseline covariates and frailty trajectory groups was provided in Table 1.

**Table 1.** Baseline characteristics of the participants according to trajectories of frailty in middle-aged and older adults from CHARLS (China Health and Retirement Longitudinal Study).

Characteristics	Class 1, low progressive (n=7208)	Class 2, moderate progressive (n=3061)	Class 3, high progressive (n=609)	*H*/χ² test	*P* value
Age, years, median (IQR)	56.0 (50.0, 62.0)	60.0 (55.0, 66.0)	63.0 (57.0, 69.0)	366.9	<.001
Sex, n (%)				189.6	<.001
Female	3410 (47.3)	1855 (60.6)	390 (64.0)		
Male	3798 (52.7)	1206 (39.4)	219 (36.0)		
Current drinking, n (%)				175.4	<.001
Yes	2759 (38.3)	813 (26.6)	132 (21.7)		
No	4449 (61.7)	2248 (73.4)	477 (78.3)		
Current smoking, n (%)				91.7	<.001
Yes	2421 (33.6)	775 (25.3)	134 (22.0)		
No	4787 (66.4)	2286 (74.7)	475 (78.0)		
Married, n (%)				110.9	<.001
Yes	6637 (92.1)	2656 (86.8)	501 (82.3)		
No	571 (7.9)	405 (13.2)	108 (17.7)		
Residence, n (%)				59.6	<.001
Rural	4457 (61.8)	2109 (68.9)	433 (71.1)		
City	2751 (38.2)	952 (31.1)	176 (28.9)		
Education, n (%)				351.2	<.001
Primary school and below	4363 (60.5)	2364 (77.2)	507 (83.3)		
Middle school	1824 (25.3)	460 (15.0)	69 (11.3)		
High school and above	1021 (14.2)	237 (7.8)	33 (5.4)		
Physical activities, n (%)				34.8	<.001
Inactivity	4500 (62.4)	1865 (60.9)	399 (65.5)		
Low-intensity activities	617 (8.6)	306 (10.0)	79 (13.0)		
Moderate activities	903 (12.5)	424 (13.9)	67 (11.0)		
Vigorous activities	1188 (16.5)	466 (15.2)	64 (10.5)		
Inpatient care, n (%)				243.2	<.001
Yes	436 (6.0)	385 (12.6)	130 (21.3)		
No	6772 (94.0)	2676 (87.4)	479 (78.7)		
Retirement, n (%)				0.1	.94
Yes	807 (11.2)	349 (11.4)	67 (11.0)		
No	6401 (88.8)	2712 (88.6)	542 (89.0)		
Social activities, n (%)				7.4	.11
0	4375 (60.7)	1938 (63.3)	385 (63.2)		
1‐2	1387 (19.2)	546 (17.8)	104 (17.1)		
≥3	1446 (20.1)	577 (18.9)	120 (19.7)		
Intellectual activities, n (%)				89.1	<.001
0	5576 (77.4)	2573 (84.1)	538 (88.3)		
1‐2	1143 (15.9)	339 (11.0)	47 (7.7)		
≥3	489 (6.7)	149 (4.9)	24 (4.0)		

### Baseline Social, Intellectual Activities Scores and Frailty Trajectories

The multinomial regression analysis (Model 2) revealed distinct associations between social/intellectual activity scores and frailty trajectory membership. For social activities, compared to the reference group (score=0): participants with 1‐2 scores showed no significant reduction in the odds of transitioning to the moderate progressive trajectory or the high progressive trajectory. Participants with ≥3 activity scores exhibited a 16% reduction in the odds of belonging to the moderate progressive trajectory (odds ratio [OR] 0.84; 95% CI 0.75‐0.94; *P*=.004), while no significant association was observed for the high progressive trajectory.

For intellectual activities, compared to the reference group (score=0): participants with 1‐2 scores demonstrated an 18% reduction in the odds of entering the moderate progressive trajectory (OR 0.82; 95% CI 0.72‐0.94; *P*=.005) and a 40% reduction in the odds of transitioning to the high progressive trajectory (OR 0.60; 95% CI 0.43‐0.83; *P*=.002). Participants with ≥3 activity scores showed even stronger protective effects: a 23% reduction in the odds for the moderate progressive trajectory (OR 0.77; 95% CI 0.63‐0.94; *P*=.01) and a 37% reduction in the odds for the high progressive trajectory (OR 0.63; 95% CI 0.40‐0.99; *P*=.04). Detailed results are presented in Table 2.

**Table 2.** Multinomial logistic regression analysis for the associations of social and intellectual activities with membership in the frailty trajectories group. Model 1 adjusted for age, sex, current drinking, current smoking; Model 2 plus additional adjustments for married, residence, education, physical activities, inpatient care, and retirement.

Variables	Model 1				Model 2			
	Moderate progressive (vs low progressive)		High progressive (vs low progressive)		Moderate progressive (vs low progressive)		High progressive (vs low progressive)	
	OR^a^ (95% CI)	*P* value	OR (95% CI)	*P* value	OR (95% CI)	*P* value	OR (95% CI)	*P* value
Social activities scores								
0	1.00 (reference)	—^b^	1.00 (reference)	—	1.00 (reference)	—	1.00 (reference)	—
1-2	0.93 (0.83‐1.05)	.26	0.94 (0.75‐1.19)	.62	0.95 (0.85‐1.07)	.43	0.92 (0.72,1.17)	.49
≥3	0.82 (0.73‐0.92)	.001	0.80 (0.64‐0.99)	.046	0.84 (0.75‐0.94)	.004	0.87 (0.69,1.09)	.23
Intellectual activities scores								
0	1.00 (reference)	—	1.00 (reference)	—	1.00 (reference)	—	1.00 (reference)	—
1-2	0.78 (0.68‐0.90)	<.001	0.54 (0.40‐0.75)	<.001	0.82 (0.72‐0.94)	.005	0.60 (0.43,0.83)	.002
≥3	0.67 (0.55‐0.82)	<.001	0.51 (0.33‐0.79)	.002	0.77 (0.63‐0.94)	.01	0.63 (0.40,0.99)	.04

a OR: odds ratio.

b Not applicable.

### Stratified Analysis

**Figure 3.**
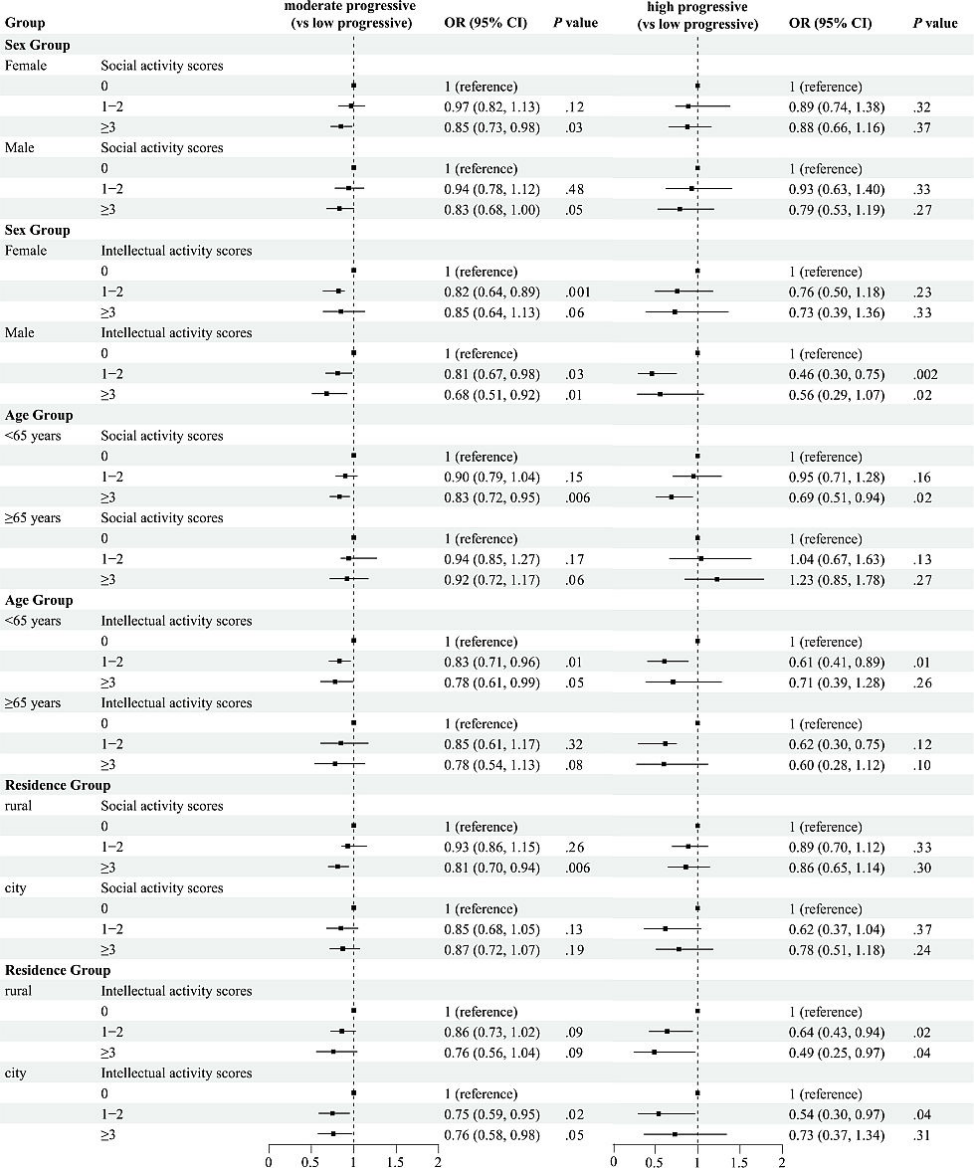
The association of intellectual activities and social activities, stratified by age group, sex group, and residence group. Odds ratio (OR), 95% CI, and *P* values test the significance of the association within each subgroup. Adjusted for age, sex, current drinking, current smoking, marital status, residence, education, physical activities, inpatient care, and retirement.

The stratified analyses are shown in Figure 3. We found that significant associations between social activities scores and frailty trajectories seem to differ with sex and age. In men, higher social activity scores had a significant effect on the moderate progression of reducing frailty, (OR 0.85; 95% CI 0.73‐0.98, *P*=.003). For women, intellectual activities demonstrated stronger protective effects against frailty progression. Women engaging in frequent intellectual activities had a 32% lower risk of entering the “moderate progressive” trajectory (OR 0.68; 95% CI 0.51‐0.92, *P*=.001). At the same time, high levels of social activities have a positive role in preventing frailty in people aged <65 years. Frequent social activity reduced the risk of “moderate progressive” (OR 0.83; 95% CI 0.72‐0.95, *P*=.05) and “high progressive” (OR 0.69; 95% CI 0.51‐0.94, *P*=.02) trajectories. In terms of the geographical distribution, rural residents showed stronger associations between social activity and favorable vulnerable trajectories(OR 0.81; 95% CI 0.70‐0.94, *P*=.006), while urban residents benefited more from structured intellectual engagement(OR 0.76; 95% CI 0.58‐0.98, *P*=.05).

### Sensitivity Analyses

In the sensitivity analyses, first, to account for potential bias introduced by participant selection, the cohort was reanalyzed after excluding 878 participants enrolled in 2013 (Table S1-S2 in Multimedia Appendix 5). Second, participants were categorized into three frailty states based on the frailty index to examine the association between distinct frailty states and engagement in social and intellectual activities. Across all sensitivity analyses, the results remained consistent, further supporting the robustness of the observed associations (Table S1-S2 in Multimedia Appendix 6).

## Discussion

### Principal Findings

In this longitudinal study, we identified three distinct frailty trajectories—low progressive, moderate progressive, and high progressive. Our findings demonstrated that frequent participation in social and intellectual activities is associated with a reduced risk of transitioning to adverse frailty trajectories. Unlike prior studies that primarily focused on frailty status or onset, our analysis captures the dynamic progression of frailty, thereby offering a longitudinal perspective on how modifiable psychosocial factors influence frailty development over time. Additionally, this study found that both social and intellectual activities can affect the frailty trajectory of different genders, different age groups, and different regions. These associations remained robust across sensitivity analyses, underscoring the potential protective role of engagement in social and intellectual activities against frailty progression. To our knowledge, this is one of the first studies to simultaneously examine the association of both social and intellectual activity frequencies with longitudinal frailty trajectories, providing a dynamic perspective on frailty progression and its modifiable psychosocial predictors.

### Association Between Social Activity and Frailty Trajectories

Consistent with previous studies [20-22], our findings demonstrate that frequent participation in social activity is significantly associated with a reduced likelihood of progression along a moderate frailty trajectory. These associations can be explained by various factors: Firstly, social activity often involves physical exertion, which enhances muscle mass, strength, balance, and cardiopulmonary function [23], thereby directly counteracting established pathways linking skeletal muscle structure and function to frailty. However, whereas previous studies largely examined social support or isolation as static risk factors, our trajectory-based approach reveals how varying levels of social activity dynamically influence frailty progression over time. Previous studies have reported that loneliness and social isolation are established risk factors for frailty [24,25]. Social activity mitigates established risk factors for frailty, such as loneliness and social isolation, by providing emotional support and improving psychological well-being. Evidence also suggests that inflammation may play a regulatory role in the progression of frailty; inflammatory responses can be exacerbated by social isolation [26-29]. Thus, increased social activities could attenuate systemic inflammation and delay frailty progression.

Notably, we observed differential effects across subgroups. Our study found that men may derive greater benefits from social activities in slowing frailty progression, potentially due to their tendency to seek support through social networks to manage stress, whereas women may prioritize educational or cultural activities [17]. Notably, the protective effect was more pronounced in participants younger than 65 years of age in the present study, possibly because the middle-aged population socializes more frequently and is more affected. Additionally, geographic differences were evident. Social activities were particularly effective in reducing frailty among rural residents, possibly due to rural China’s social structure and residential distribution, which leads to a strong reliance on kinship networks. These networks provide instrumental help and reduce loneliness through the incorporation of physical labor in agriculture-based social activity.

### Association Between Intellectual Activity and Frailty Trajectories

Our findings corroborate earlier observational studies linking intellectual activity to a lower risk of frailty [11], but extend them by demonstrating a longitudinal and dose–response relationship between intellectual engagement and frailty trajectory. However, prior research has not adequately addressed the longitudinal effects of social and intellectual activities on frailty trajectories. Frailty often coexists with cognitive impairment [30]. Lack of intellectual activity increases the risks of cognitive impairment [31], which in turn creates a higher risk of frailty occurrences. In a cross-sectional study, Wang et al [32] found that engaging in intellectual activity in later life was associated with a lower prevalence of frailty, particularly among older adult women. This aligns with our observation in females that intellectual activity exerts a stronger protective effect against frailty progression in females. Furthermore, urban residents benefited more from intellectual activities; this may be due to urban areas with better-developed public infrastructure and information accessibility may facilitate cognitive stimulation through intellectual engagement.

Frailty has evolved from the initial concept to a complex, multidimensional bio-psycho-social syndrome [33]. Compared to previous interventions that have focused on physical activity and nutritional strategies, our findings highlight the importance of integrating social and psychological strategies. Based on the results of our study showing a significant association between participation in social activity and intellectual activity and reduced vulnerability development, this encourages the inclusion of frailty interventions in future social activity and intellectual activity intervention strategies in middle-aged and older adults. Specifically, tailored interventions should be implemented. For men, social activities should be actively promoted to delay the development process of frailty, while women should carry out a variety of learning skills from the perspective of intellectual activities. From the age group, we advocate intervention in the development of the frailty trajectory early in middle age, which can effectively prevent and reduce the development of frailty. In rural areas, enhancing public infrastructure could amplify the benefits of intellectual activities, whereas urban communities should prioritize social activity programs to address isolation risks. Furthermore, if social and intellectual activity are performed simultaneously and combined with physical exercise, it may have a positive impact on maintaining the normal operation of the physiological system, thus delaying the progression of cognitive fragility [34-36].

### Strengths and Limitations of This Study

To our knowledge, this is the first study to investigate the relationship between social and intellectual activity and frailty trajectory. The strength of this study is that we used a relatively large nationally representative cohort of Chinese middle-aged and elderly people to evaluate the influence of social activity and intellectual activity on frailty trajectory, and we found that frequent intellectual activity was associated with a lower frailty trajectory, suggesting that we provide a new direction for the management and prevention of frailty. Furthermore, state-of-the-art statistical models were used to fit frailty development trajectories. This approach helps to identify groups of individuals who have experienced similar levels and patterns over time, and more helps to identify people with similar frailty trajectories, thus rationing the intervention for the population.

However, limitations should be acknowledged. First, despite rigorous adjustment, residual confounding from unmeasured factors cannot be ruled out. Specifically, more granular data on socioeconomic status, nutritional status were not available. These factors may influence both activity participation and frailty risk. Second, self-reported activity frequencies are susceptible to recall bias, though nondifferential misclassification would likely attenuate observed associations. Third, the generalizability of our findings to other ethnic or cultural contexts may be limited. The CHARLS cohort, while nationally representative of middle-aged and older Chinese adults, has distinct sociodemographic characteristics that may differ from populations in Western countries.

### Conclusion

This study provides novel evidence that social and intellectual activities are independently associated with favorable frailty trajectories in middle-aged and older adults. The sex- and age-specific effects highlight the need for tailored interventions—promoting social engagement in men and intellectual stimulation in women, particularly in younger populations. Furthermore, future research should investigate whether interventions that simultaneously combine social, intellectual, and physical activity could have synergistic effects in maintaining physiological system integrity and thus delaying frailty progression, building on existing evidence.

## Supplementary material

10.2196/80152 Multimedia Appendix 1 Comparison of baseline characteristics between the analytical sample and participants lost to follow-up.

10.2196/80152 Multimedia Appendix 2 List of items included in the frailty index in this study.

10.2196/80152 Multimedia Appendix 3 List of items included in the social and intellectual activity in this study.

10.2196/80152 Multimedia Appendix 4 Fit statistics for global frailty group trajectories in middle-aged and older adults from CHARLS (China Health and Retirement Longitudinal Study).

10.2196/80152 Multimedia Appendix 5 Sensitivity analysis 1.

10.2196/80152 Multimedia Appendix 6 Sensitivity analysis 2.

## Abbreviations

ADL: Activities of Daily Living
BIC: Bayesian Information Criterion
CHARLS: China Health and Retirement Longitudinal Study
FI: Frailty Index
GBTM: Group-Based Trajectory Modeling
IADL: Instrumental Activities of Daily Living
OR: odds ratio

## References

[1] Dent E, Martin FC, Bergman H, Woo J, Romero-Ortuno R, Walston JD (2019). Management of frailty: opportunities, challenges, and future directions. Lancet.

[2] Hoogendijk EO, Afilalo J, Ensrud KE, Kowal P, Onder G, Fried LP (2019). Frailty: implications for clinical practice and public health. Lancet.

[3] Collard RM, Boter H, Schoevers RA, Oude Voshaar RC (2012). Prevalence of frailty in community-dwelling older persons: a systematic review. J Am Geriatr Soc.

[4] Ofori-Asenso R, Chin KL, Mazidi M (2019). Global incidence of frailty and prefrailty among community-dwelling older adults: a systematic review and meta-analysis. JAMA Netw Open.

[5] Walston J, Buta B, Xue QL (2018). Frailty screening and interventions: considerations for clinical practice. Clin Geriatr Med.

[6] Woolford SJ, Sohan O, Dennison EM, Cooper C, Patel HP (2020). Approaches to the diagnosis and prevention of frailty. Aging Clin Exp Res.

[7] Ding YY, Kuha J, Murphy M (2017). Multidimensional predictors of physical frailty in older people: identifying how and for whom they exert their effects. Biogerontology.

[8] Fustinoni S, Santos-Eggimann B, Henchoz Y (2022). Trajectories of phenotypical frailty over a decade in young-old community-dwelling adults: results from the Lc65+ study. J Epidemiol Community Health.

[9] Jin Y, Yu R, Si H (2022). Effects of social support on frailty trajectory classes among community-dwelling older adults: The mediating role of depressive symptoms and physical activity. Geriatr Nurs.

[10] Peek MK, Howrey BT, Ternent RS, Ray LA, Ottenbacher KJ (2012). Social support, stressors, and frailty among older Mexican American adults. The Journals of Gerontology Series B: Psychological Sciences and Social Sciences.

[11] Huang Y, Guo X, Du J, Liu Y (2021). Associations between intellectual and social activities with frailty among community-dwelling older adults in China: a prospective cohort study. Front Med.

[12] Zhao Y, Hu Y, Smith JP, Strauss J, Yang G (2014). Cohort profile: the China Health and Retirement Longitudinal Study (CHARLS). Int J Epidemiol.

[13] Searle SD, Mitnitski A, Gahbauer EA, Gill TM, Rockwood K (2008). A standard procedure for creating a frailty index. BMC Geriatr.

[14] Yin JH, Zeng YB, Zhou Z, Fang Y (2018). Study on the status of frailty and related determinants among the elderly in China. Zhonghua Liu Xing Bing Xue Za Zhi.

[15] Wang X, Chen Z, Li Z (2020). Association between frailty and risk of fall among diabetic patients. Endocr Connect.

[16] Nagin DS, Odgers CL (2010). Group-based trajectory modeling in clinical research. Annu Rev Clin Psychol.

[17] Reid N, Young A, Shafiee Hanjani L, Hubbard RE, Gordon EH (2022). Sex-specific interventions to prevent and manage frailty. Maturitas.

[18] Bellelli F, Consorti E, Hettiarachchige TMK (2023). Relationship among age, education and frailty in older persons. J Frailty Aging.

[19] Xie X, Que J, Sun L, Sun T, Yang F (2025). Association between urbanization levels and frailty among middle-aged and older adults in China: evidence from the CHARLS. BMC Med.

[20] Makizako H, Shimada H, Doi T (2018). Social frailty leads to the development of physical frailty among physically non-frail adults: a four-year follow-up longitudinal cohort study. Int J Environ Res Public Health.

[21] Chon D, Lee Y, Kim J, Lee K eun (2018). The association between frequency of social contact and frailty in older people: Korean Frailty and Aging Cohort Study (KFACS). J Korean Med Sci.

[22] Chong EY, Lim AHS, Mah FCY, Yeo LHW, Ng ST, Yi H (2022). Assessing the psychosocial dimensions of frailty among older adults in Singapore: a community-based cross-sectional study. BMJ Open.

[23] Angulo J, El Assar M, Álvarez-Bustos A, Rodríguez-Mañas L (2020). Physical activity and exercise: strategies to manage frailty. Redox Biol.

[24] Gale CR, Westbury L, Cooper C (2018). Social isolation and loneliness as risk factors for the progression of frailty: the English Longitudinal Study of Ageing. Age Ageing.

[25] Kojima G, Taniguchi Y, Aoyama R, Tanabe M (2022). Associations between loneliness and physical frailty in community-dwelling older adults: A systematic review and meta-analysis. Ageing Res Rev.

[26] Ferrucci L, Fabbri E (2018). Inflammageing: chronic inflammation in ageing, cardiovascular disease, and frailty. Nat Rev Cardiol.

[27] Soysal P, Stubbs B, Lucato P (2016). Inflammation and frailty in the elderly: a systematic review and meta-analysis. Ageing Res Rev.

[28] Smith KJ, Gavey S, RIddell NE, Kontari P, Victor C (2020). The association between loneliness, social isolation and inflammation: A systematic review and meta-analysis. Neurosci Biobehav Rev.

[29] Matthews T, Rasmussen LJH, Ambler A (2024). Social isolation, loneliness, and inflammation: a multi-cohort investigation in early and mid-adulthood. Brain Behav Immun.

[30] Yuan Y, Peng C, Burr JA, Lapane KL (2023). Frailty, cognitive impairment, and depressive symptoms in Chinese older adults: an eight-year multi-trajectory analysis. BMC Geriatr.

[31] Lam LCW, Ong PA, Dikot Y (2015). Intellectual and physical activities, but not social activities, are associated with better global cognition: a multi-site evaluation of the cognition and lifestyle activity study for seniors in Asia (CLASSA). Age Ageing.

[32] Wang X, Lu Y, Li C (2020). Associations of lifestyle activities and a heathy diet with frailty in old age: a community-based study in Singapore. Aging (Milano).

[33] Cohen CI, Benyaminov R, Rahman M, Ngu D, Reinhardt M (2023). Frailty: a multidimensional biopsychosocial syndrome. Med Clin North Am.

[34] Aartsen MJ, Smits CHM, van Tilburg T, Knipscheer K, Deeg DJH (2002). Activity in older adults: cause or consequence of cognitive functioning? A longitudinal study on everyday activities and cognitive performance in older adults. J Gerontol B Psychol Sci Soc Sci.

[35] Cheong CY, Nyunt MSZ, Gao Q (2020). Risk factors of progression to frailty: findings from the Singapore Longitudinal Ageing Study. J Nutr Health Aging.

[36] Jia J, Zhao T, Liu Z (2023). Association between healthy lifestyle and memory decline in older adults: 10 year, population based, prospective cohort study. BMJ.

[37] China Health and Retirement Longitudinal Study (CHARLS).

