# Supplementary material for: The Dynamic Associations of Social and Intellectual Activity With Frailty Trajectory in Middle-Aged and Older Adults in China: Nationwide Longitudinal Study
Source: JMIR Aging. 2025 Dec 15;8:e80152. doi: 10.2196/80152 (PMC12704913; doi:10.2196/80152)
Supplement: Multimedia Appendix 1 [file aging-v8-e80152-s001.docx]

**Multimedia Appendix 1:**

A comparison of baseline characteristics between the analytical sample (n = 10,878) and the simulated sample of participants lost to follow-up (n = 4,548) was conducted to assess potential selection bias. The results are presented in ***Table S1*** Statistically significant differences were observed in all baseline characteristics examined (all P-values < .05). Participants lost to follow-up were, on average, older and had a higher prevalence of factors associated with poorer health and socioeconomic status. Specifically, the education group had a higher proportion of individuals with primary school education or below, rural residence, and a history of inpatient care. They also reported lower engagement in health-promoting behaviors, as evidenced by a lower prevalence of current drinking and smoking, and significantly less frequent participation in both social and intellectual activities.

**Table S1** Comparison of Baseline Characteristics Between the Analytical Sample and Participants Lost to Follow-up

| Characteristics | Analytical Sample (n = 10,878) | Lost-to-Follow-up Sample (n = 4,548) | *P* value |
| --- | --- | --- | --- |
| Age, years | 58.0 (51.0, 64.0) | 60.0 (54.0, 67.0) | <0.001 |
| Female, n (%) | 5655 (52.0) | 2450 (53.9) | 0.032 |
| Current Drink n (%) | 3704 (34.1) | 1420 (31.2) | <0.001 |
| Current Smoke n (%) | 3266 (30.0) | 1250 (27.5) | 0.001 |
| Married n (%) | 9794 (90.0) | 3980 (87.5) | <0.001 |
| Rural n (%) | 6999 (64.3) | 3050 (67.1) | 0.001 |
| Education n (%) |  |  | <0.001 |
| Primary school and below | 7231 (66.5) | 3250 (71.5) |  |
| Middle school | 2351 (21.6) | 850 (18.7) |  |
| High school and above | 1288 (11.9) | 448 (9.8) |  |
| Physical Activities n (%) |  |  | <0.001 |
| Inactivity | 6764 (62.2) | 2950 (64.9) |  |
| Low-Intensity Activities | 1002 (9.2) | 450 (9.9) |  |
| Moderate Activities | 1394 (12.8) | 500 (11.0) |  |
| Vigorous Activities | 1718 (15.8) | 648 (14.2) |  |
| Have Inpatient Care | 950 (8.7) | 500 (11.0) | <0.001 |
| Have Retirement | 9484 (88.7) | 3980 (87.5) | 0.027 |
| Social activity n (%) |  |  | <0.001 |
| 0 | 6698(61.6) | 2950 (64.9) |  |
| 1-2 | 2037(18.7) | 800 (17.6) |  |
| ≥3 | 2143(19.7) | 798 (17.5) |  |
| Intellectual activity n (%) |  |  | <0.001 |
| 0 | 8687(79.8) | 3750 (82.5) |  |
| 1-2 | 1529(14.1) | 548 (12.0) |  |
| ≥3 | 662(6.1) | 250 (5.5) |  |
