# Supplementary material for: The Dynamic Associations of Social and Intellectual Activity With Frailty Trajectory in Middle-Aged and Older Adults in China: Nationwide Longitudinal Study
Source: JMIR Aging. 2025 Dec 15;8:e80152. doi: 10.2196/80152 (PMC12704913; doi:10.2196/80152)
Supplement: Multimedia Appendix 2 [file aging-v8-e80152-s002.docx]

**Multimedia Appendix 2:**

In this study, for binary variables, the responses were coded as Yes = 1 and No =0. For ordinal variables with four levels, such as activities of daily living (ADLs), they were converted into binary variables with the following scoring criteria: 'Yes, I have difficulty and need help' or 'I can't do it' = 1, others =0. For ordinal variables with five levels, such as self-reported health status, they are also converted into binary variables with the following scoring criteria: Poor or Very poor = 1, others =0. The depression status is measured using the Centre for Epidemiological Studies Depression Scale (CES-D). A CES-D score of 10 or higher = 1, not depressed = 0. ***See Table S2*** for details.

**Table S2**. List of items included in the frailty index in this study

| **No** | **Variables** | **Values** |
| --- | --- | --- |
| 1 | Self-reported health | Poor/very poor=1, others=0 |
| 2 | Hypertension | Yes=1, no=0 |
| 3 | Diabetes/high blood sugar | Yes=1, no=0 |
| 4 | Cancer | Yes=1, no=0 |
| 5 | Chronic lung diseases | Yes=1, no=0 |
| 6 | Heart attack | Yes=1, no=0 |
| 7 | Stroke | Yes=1, no=0 |
| 8 | Emotional, nervous, or psychiatric problems | Yes=1, no=0 |
| 9 | Arthritis | Yes=1, no=0 |
| 10 | Dyslipidaemia | Yes=1, no=0 |
| 11 | Liver diseases | Yes=1, no=0 |
| 12 | Kidney | Yes=1, no=0 |
| 13 | Stomach | Yes=1, no=0 |
| 14 | Asthma | Yes=1, no=0 |
| 15 | Memory-related diseases | Yes=1, no=0 |
| 16 | Visual problem | Yes=1, no=0 |
| 17 | Hearing problem | Yes=1, no=0 |
| 18 | Physical disabilities | Yes=1, no=0 |
| 19 | Fall-Off | Yes=1, no=0 |
| 20 | Difficulty with walking at least 100m | Yes, I have difficulty and need help/I can not do it=1,  others=0 |
| 21 | Difficulty with getting up from a chair after sitting for a long period | Yes, I have difficulty and need help/I can not do it=1,  others=0 |
| 22 | Difficulty with climbing stairs | Yes, I have difficulty and need help/I can not do it=1,  others=0 |
| 23 | Difficulty with stooping/kneeling/crouching | Yes, I have difficulty and need help/I can not do it=1,  others=0 |
| 24 | Difficulty with extending arms | Yes, I have difficulty and need help/I can not do it=1,  others=0 |
| 25 | Difficulty with lifting weights over 5kg | Yes, I have difficulty and need help/I can not do it=1,  others=0 |
| 26 | Difficulty with picking up a small coin from a table | Yes, I have difficulty and need help/I can not do it=1,  others=0 |
| 27 | Difficulty with dressing | Yes, I have difficulty and need help/I can not do it=1,  others=0 |
| 28 | Difficulty with bathing | Yes, I have difficulty and need help/I can not do it=1,  others=0 |
| 29 | Difficulty with eating | Yes, I have difficulty and need help/I can not do it=1,  others=0 |
| 30 | Difficulty with getting into or out of bed | Yes, I have difficulty and need help/I can not do it=1,  others=0 |
| 31 | Difficulty with using toilet | Yes, I have difficulty and need help/I can not do it=1,  others=0 |
| 32 | Difficulty with controlling urination and defecation | Yes, I have difficulty and need help/I can not do it=1,  others=0 |
| 33 | Difficulty with doing household chores | Yes, I have difficulty and need help/I can not do it=1,  others=0 |
| 34 | Difficulty with preparing hot meals | Yes, I have difficulty and need help/I can not do it=1,  others=0 |
| 35 | Difficulty with shopping | Yes, I have difficulty and need help/I can not do it=1,  others=0 |
| 36 | Difficulties with making phone calls | Yes, I have difficulty and need help/I can not do it=1,  others=0 |
| 37 | Difficulty with taking medications | Yes, I have difficulty and need help/I can not do it=1,  others=0 |
| 38 | Depression (measured CES-D^a^) | Depressed (scores of CES-D ≥ 10)=1, not depressed=0 |

Note. a. CES-D = Centre for Epidemiological Studies Depression Scale.^1、^

1. Andresen EM, Malmgren JA, Carter WB, Patrick DL. Screening for depression in well older adults: Evaluation of a short form of the CES-D. *American journal of preventive medicine*. 1994;10(2):77-84. doi:10.1016/S0749-3797(18)30622-6
