# Supplementary material for: The Dynamic Associations of Social and Intellectual Activity With Frailty Trajectory in Middle-Aged and Older Adults in China: Nationwide Longitudinal Study
Source: JMIR Aging. 2025 Dec 15;8:e80152. doi: 10.2196/80152 (PMC12704913; doi:10.2196/80152)
Supplement: Multimedia Appendix 3 [file aging-v8-e80152-s003.docx]

**Multimedia Appendix 3:**

In this study, participants were asked whether they had engaged in social activities and intellectual activities in the past month, According to the types of activities that participants participate in, those who participate in three or more activities are considered to be regular participants, those who participate in 1-2 activities are considered to be infrequent participants, and those who do not participate in all four activities are considered to be non-participants. See ***Table S3*** for details.

**Table S3.** List of items included in the social and intellectual activity in this study

| **Items** | **Variables** | **Values** |
| --- | --- | --- |
| Social activities | Interacted with friends | ≥3 (frequent participation)  1–2 (non-regular participation)  0 (no participation) |
|  | Went to a sport, social, or other kind of club |  |
|  | Took part in a community-related organization |  |
|  | Done voluntary or charity work |  |
| Intellectual activity | Played Ma-jong, played chess, played cards, or went to community club | ≥3 (frequent participation)  1–2 (non-regular participation)  0 (no participation) |
|  | Attended an educational or training course |  |
|  | Stock investment |  |
|  | Used the Internet |  |
