# Supplementary material for: The Dynamic Associations of Social and Intellectual Activity With Frailty Trajectory in Middle-Aged and Older Adults in China: Nationwide Longitudinal Study
Source: JMIR Aging. 2025 Dec 15;8:e80152. doi: 10.2196/80152 (PMC12704913; doi:10.2196/80152)
Supplement: Multimedia Appendix 4 [file aging-v8-e80152-s004.docx]

**Multimedia Appendix 4:**

The optimal trajectories groups are expected to exhibit the following characteristics to the greatest extent possible:the smaller absolute Bayesian Information Criteria (BIC) value; the bigger change in BIC; the value of the Average Posterior Probability (APP) > 0.7; Odds of Correct Classification (OCC) > 5; Proportions per class ≥ 5%. See ***Table S4*** for details.

**Table S4** Fit statistics for global frailty group trajectories in middle-aged and older adults from CHARLS

| Fit statistic | Number of classes | | | | |
| --- | --- | --- | --- | --- | --- |
|  | 1 | 2 | **3** | 4 | 5 |
| BIC* | 33780.94 | 43465.54 | **32353.79** | 48648.90 | 40971.74 |
| AIC* | 33792.57 | 43492.16 | **32390.26** | 48706.57 | 41030.0 |
| Class proportion | Class1,100% | Class1,79.13%  Class2,20.87% | **Class1,65.46%**  **Class2,28.66%**  **Class3,5.89%** | Class1,51.89%  Class2,32.14%  Class3,13.08%  Class4,2.89% | Class1,49.93%  Class2,32.38%  Class3,9.37%  Class4,5.56%  Class5,2.77% |
| Entropy | - | 0.87 | **0.85** | 0.78 | 0.72 |
| Avepp | 1.00 | 0.97  0.92 | **0.95**  **0.88**  **0.93** | 0.89  0.80  0.86  0.92 | 0.81  0.70  0.35  0.34  0.18 |

^APP average posterior probabilities^
