# Supplementary material for: The Dynamic Associations of Social and Intellectual Activity With Frailty Trajectory in Middle-Aged and Older Adults in China: Nationwide Longitudinal Study
Source: JMIR Aging. 2025 Dec 15;8:e80152. doi: 10.2196/80152 (PMC12704913; doi:10.2196/80152)
Supplement: Multimedia Appendix 5 [file aging-v8-e80152-s005.docx]

**Multimedia Appendix 5:**

**Sensitivity analysis 1：**A logical regression analysis of population baseline and frailty trajectory and social and intellectual activity was performed after excluding 878 subjects enrolled in 2013. After excluding 878 participants, the age of participants in the population gradually increased, and the proportion of women, those who had not smoked or drunk alcohol in the past, those who lived in rural areas, those who were married, those with primary school education or below, those who reported physical inactivity, and those who had retired gradually increased (*P* <.001). See ***Table S1-S2*** for details.

**Table S1** Baseline characteristics of participants in the CHARLS trajectory of frailty in middle age (excluding 878 new participants included in 2013)

| Characteristics | Class 1, low progressive (n = 6768) | Class 2, moderate progressive (n = 2841) | Class 3, high progressive (n = 527) | *H*/χ² test | *P* value |
| --- | --- | --- | --- | --- | --- |
| Age, years | 56.0 (50.0, 62.0) | 60.0 (55.0, 66.0) | 62.5 (57.0, 69.0) | 510.7 | <.001 |
| Gender, n (%) |  |  |  | 189.6 | <.001 |
| Female | 3410 (47.3) | 1855 (60.6) | 390 (64.0) |  |  |
| Male | 3798 (52.7) | 1206 (39.4) | 219 (36.0) |  |  |
| Current Drink n (%) |  |  |  | 175.4 | <.001 |
| Yes | 2759 (38.3) | 813 (26.6) | 132 (21.7) |  |  |
| No | 4449 (61.7) | 2248 (73.4) | 477 (78.3) |  |  |
| Current Smoke n (%) |  |  |  | 91.7 | <.001 |
| Yes | 2421 (33.6) | 775 (25.3) | 134 (22.0) |  |  |
| No | 4787 (66.4) | 2286 (74.7) | 475 (78.0) |  |  |
| Married n (%) |  |  |  | 110.9 | <.001 |
| Yes | 6637 (92.1) | 2656 (86.8) | 501 (82.3) |  |  |
| No | 571 (7.9) | 405 (13.2) | 108 (17.7) |  |  |
| Residence n (%) |  |  |  | 59.6 | <.001 |
| Rural | 4457 (61.8) | 2109 (68.9) | 433 (71.1) |  |  |
| City | 2751 (38.2) | 952 (31.1) | 176 (28.9) |  |  |
| Education n (%) |  |  |  | 351.2 | <.001 |
| Primary school and below | 4363 (60.5) | 2364 (77.2) | 507 (83.3) |  |  |
| Middle school | 1824 (25.3) | 460 (15.0) | 69 (11.3) |  |  |
| High school and above | 1021 (14.2) | 237 (7.8) | 33 (5.4) |  |  |
| Physical Activities n (%) |  |  |  | 34.8 | <.001 |
| Inactivity | 4500 (62.4) | 1865 (60.9) | 399 (65.5) |  |  |
| Low-Intensity Activities | 617 (8.6) | 306 (10.0) | 79 (13.0) |  |  |
| Moderate Activities | 903 (12.5) | 424 (13.9) | 67 (11.0) |  |  |
| Vigorous Activities | 1188 (16.5) | 466 (15.2) | 64 (10.5) |  |  |
| Inpatient Care n (%) |  |  |  | 243.2 | <.001 |
| Yes | 436 (6.0) | 385 (12.6) | 130 (21.3) |  |  |
| No | 6772 (94.0) | 2676 (87.4) | 479 (78.7) |  |  |
| Retirement n (%) |  |  |  | 0.1 | .938 |
| Yes | 807 (11.2) | 349 (11.4) | 67 (11.0) |  |  |
| No | 6401 (88.8) | 2712 (88.6) | 542 (89.0) |  |  |
| Social Activities n (%) |  |  |  | 7.4 | .113 |
| 0 | 4375(60.7) | 1938(63.3) | 385(63.2) |  |  |
| 1-2 | 1387(19.2) | 546(17.8) | 104(17.1) |  |  |
| ≥3 | 1446(20.1) | 577(18.9) | 120(19.7) |  |  |
| Intellectual Activities n (%) |  |  |  | 89.1 | <.001 |
| 0 | 5576(77.4) | 2573(84.1) | 538(88.3) |  |  |
| 1-2 | 1143(15.9) | 339(11.0) | 47(7.7) |  |  |
| ≥3 | 489(6.7) | 149(4.9) | 24(4.0) |  |  |

The results of the sensitivity analysis show that for social activities, compared to the reference group (score = 0): participants with scores ≥3 have a 14% lower chance of belonging to the moderate progression trajectory (OR = 0.86; 95% CI: 0.76–0.97; *P* = .017), while no significant association was observed for the high progression trajectory. For intellectual activities, compared to the reference group (score = 0): participants with scores 1-2 have a 16% lower chance of transitioning to the moderate progression trajectory and a 41% lower chance of transitioning to the high progression trajectory (OR = 0.84; 95% CI: 0.73–0.96; *P* = .011) and (OR = 0.59; 95% CI: 0.42–0.83; *P* = .002), respectively. Participants with scores ≥3 have a 20% lower chance of belonging to the moderate progression trajectory (OR = 0.80; 95% CI: 0.65–0.98; *P* = .029), while no significant association was observed for the high progression trajectory

**Table S2** Multinomial logistic regression analysis for the associations of social and intellectual activities with the membership to frailty trajectories group(excluding 878 new participants included in 2013)

|  | Model 2 | | | | |
| --- | --- | --- | --- | --- | --- |
|  | moderate progressive (vs low progressive) | |  | high progressive (vs low progressive) | |
|  | OR (95% CI) | *P* value |  | OR (95% CI) | *P* value |
| Social activity scores |  |  |  |  |  |
| 0 | 1.00 (reference) |  |  | 1.00 (reference) |  |
| 1-2 | 0.96(0.85,1.08) | .517 |  | 1.05(0.83,1.36) | .646 |
| ≥3 | 0.86(0.76,0.97) | .017 |  | 0.82(0.64,1.05) | .110 |
| Intellectual activity scores |  |  |  |  |  |
| 0 | 1.00 (reference) |  |  | 1.00 (reference) |  |
| 1-2 | 0.84(0.73,0.96) | .011 |  | 0.59(0.42,0.83) | .002 |
| ≥3 | 0.80(0.65,0.98) | .029 |  | 0.64(0.40,1.01) | .059 |

OR odds ratio, 95% CI 95% confidence intervals.

Model 2 adjusted for age, sex, current drinking, current smoking married, residence, education, physical activities, inpatient care, retirement.
