# Supplementary material for: The Dynamic Associations of Social and Intellectual Activity With Frailty Trajectory in Middle-Aged and Older Adults in China: Nationwide Longitudinal Study
Source: JMIR Aging. 2025 Dec 15;8:e80152. doi: 10.2196/80152 (PMC12704913; doi:10.2196/80152)
Supplement: Multimedia Appendix 6 [file aging-v8-e80152-s006.docx]

**Multimedia Appendix 6:**

**Sensitivity analysis 2：**The sensitivity analysis,divide the three frailty states according to the frailty index, and analyze the relationship between different frailty states and social and intellectual activities. As the degree of frailty increased, the age of participants in the population gradually increased, and the proportion of women, those who had not smoked or drunk alcohol in the past, those who lived in rural areas, those who were married, those with primary school education or below, those who reported physical inactivity, and those who had retired gradually increased (*P* <0.001)See ***Table S1-S2*** for details.

**Table S1** Baseline characteristics of participants in the CHARLS trajectory of frailty in middle age (Different states of weakness are distinguished)

| Characteristics | Strong  (n= 6370) | Pre-frailty  (n=3836) | Frailty  (n = 672) | *H*/χ² test | *P* value |
| --- | --- | --- | --- | --- | --- |
| Age, years | 56.0 (50.0, 62.0) | 59.0 (54.0, 65.0) | 63.0 (57.0, 69.0) | 366.9 | <.001 |
| Gender, n (%) |  |  |  | 144.2 | <.001 |
| Female | 3026 (47.5) | 2183 (56.9) | 446 (66.4) |  |  |
| Male | 3344 (52.5) | 1653 (43.1) | 226 (33.6) |  |  |
| Current Drink n (%) |  |  |  | 128.8 | <.001 |
| Yes | 2410 (37.8) | 1164 (30.3) | 130 (19.3) |  |  |
| No | 3960 (62.2) | 2672 (69.7) | 542 (80.7) |  |  |
| Current Smoke n (%) |  |  |  | 79.4 | <.001 |
| Yes | 2129 (33.4) | 1074 (28.0) | 127 (18.9) |  |  |
| No | 4241 (66.6) | 2762 (72.0) | 545 (81.1) |  |  |
| Married n (%) |  |  |  | 67.4 | <.001 |
| Yes | 5847 (91.8) | 3386 (88.3) | 561 (83.5) |  |  |
| No | 523 (8.2) | 450 (11.7) | 111 (16.5) |  |  |
| Residence n (%) |  |  |  | 63.5 | <.001 |
| Rural | 3916 (61.5) | 2590 (67.5) | 493 (73.4) |  |  |
| Urban | 2454 (38.5) | 1246 (32.5) | 179 (26.6) |  |  |
| Education n (%) |  |  |  | 308.1 | <.001 |
| Primary school and below | 3824 (60.0) | 2856 (77.5) | 554 (82.4) |  |  |
| Middle school | 1622 (25.5) | 647 (16.8) | 84 (12.5) |  |  |
| High school and above | 924 (14.5) | 333 (8.7) | 34 (5.1) |  |  |
| Physical Activities n (%) |  |  |  | 20.6 | .002 |
| Inactivity | 3948 (62.0) | 2394 (62.4) | 422 (62.8) |  |  |
| Low-Intensity Activities | 538 (8.4) | 383 (10.0) | 81 (12.1) |  |  |
| Moderate Activities | 829 (13.0) | 482 (12.6) | 83 (12.3) |  |  |
| Vigorous Activities | 1055 (16.6) | 577 (15.0) | 86 (12.8) |  |  |
| Inpatient Care n (%) |  |  |  | 325.1 | <.001 |
| Yes | 324 (5.1) | 478 (12.5) | 149 (22.2) |  |  |
| No | 6046 (94.9) | 3358 (87.5) | 523 (77.8) |  |  |
| Retirement n (%) |  |  |  | 4.5 | .104 |
| Yes | 705 (11.1) | 456 (11.9) | 62 (9.2) |  |  |
| No | 5665 (88.9) | 3380 (88.1) | 610 (90.8) |  |  |
| Social Activities n (%) |  |  |  |  | .007 |
| 0 | 3843(60.3) | 2407(62.7) | 448(66.7) | 14.1 |  |
| 1-2 | 1222(19.2) | 705(18.4) | 110(16.3) |  |  |
| ≥3 | 1305(20.5) | 724(18.9) | 114(17.0) |  |  |
| Intellectual Activities n (%) |  |  |  |  | <.001 |
| 0 | 4913(77.1) | 3177(82.8) | 597(88.8) | 84.5 |  |
| 1-2 | 1013(15.9) | 461(12.0) | 55(8.2) |  |  |
| ≥3 | 444(7.0) | 198(5.2) | 20(3.0) |  |  |

The results show that for social activities, compared to the control group (score = 0): participants with scores ≥3 have a 16% lower likelihood of being in the pre-frailty stage (OR = 0.84; 95% CI: 0.75–0.94; *P* = .002) and a 21% lower likelihood of being in frailty (OR = 0.79; 95% CI: 0.61–0.98; *P* = .030). For intellectual activities, compared to the control group (score = 0): participants with scores 1-2 and those with scores ≥3 have a 15% lower likelihood of transitioning to the pre-frailty stage (OR = 0.85; 95% CI: 0.75–0.96; *P* = .009) and a 23% lower likelihood of being in frailty (OR = 0.77; 95% CI: 0.64–0.92; *P* = .006), respectively.

**Table S2** The relationship between different frailty states and social and intellectual activity

|  | Model 2 | | | | |
| --- | --- | --- | --- | --- | --- |
|  | pre-frailty (vs strong) | |  | frailty (vs strong) | |
|  | OR (95% CI) | *P* value |  | OR (95% CI)* | *P* value |
| Social activities scores |  |  |  |  |  |
| 0 | 1.00 (reference) |  |  | 1.00 (reference) |  |
| 1-2 | 0.98(0.88,1.09) | .237 |  | 0.87(0.68,1.10) | .248 |
| ≥3 | 0.84(0.75,0.94) | .002 |  | 0.79(0.61,0.98) | .030 |
| Intellectual activities scores |  |  |  |  |  |
| 0 | 1.00 (reference) |  |  | 1.00 (reference) |  |
| 1-2 | 0.85(0.75,0.96) | .009 |  | 0.78(0.57,1.05) | .098 |
| ≥3 | 0.77(0.64,0.92) | .006 |  | 0.65(0.40,1.06) | .086 |

OR odds ratio, 95% CI 95% confidence intervals.

Model 2 adjusted for age, sex, current drinking, current smoking married, residence, education, physical activities, inpatient care, retirement.
